# Computer-controlled apparatus for automated development of continuous flow methods

**DOI:** 10.1155/S1463924689000453

**Published:** 1989

**Authors:** Peter D. Wentzell, Michael J. Hatton, Paul M. Shiundu, Ronald M. Ree, Adrian P. Wade, D. Betteridge, Timothy J. Sly

**Affiliations:** 1 Department of Chemistry University of British Columbia Vancouver V6T 1Y6 Canada; 2 BP Research Centre Chertsey Road Sunbury-on-Thames TW16 7LM UK; 3 Grace Service Chemicals Foundry Lane Widnes WA8 8UD UK; 4 Department of Chemistry Dalhousie University Halifax Nova Scotia B3H 4J3 Canada

## Abstract

An automated apparatus to assist in the development of analytical continuous flow methods is described. The system is capable of controlling and monitoring a variety of pumps, valves, and detectors through an IBM PC-AT compatible computer. System components consist of two types of peristaltic pumps (including a multiple pump unit), syringe pumps, electrically and pneumatically actuated valves, and an assortment of spectrophotometric and electrochemical detectors. Details of the interface circuitry are given where appropriate. To demonstrate the utility of the system, an automatically generated response surface is presented for the flow injection determination of iron(II) by its reaction with 1,10-phenanthroline.


Journal of Automatic Chemistry Vol. 11, No. 5 (September–October 1989), pp. 227–234

### Computer-controlled apparatus for automated development of continuous flow methods

**Peter D. Wentzell†, Michael J. Hatton, Paul M. Shiundu, Ronald M. Ree, Adrian P. Wade‡**

*Department of Chemistry, University of British Columbia, Vancouver, Canada V6T 1Y6*

**D. Betteridge**

*BP Research Centre, Chertsey Road, Sunbury-on-Thames TW16 7LM, UK*

**and Timothy J. Sly**

*Grace Service Chemicals, Foundry Lane, Widnes WA8 8UD, UK*

*An automated apparatus to assist in the development of analytical continuous flow methods is described. The system is capable of controlling and/or monitoring a variety of pumps, valves, and detectors through an IBM PC-AT compatible computer. System components consist of two types of peristaltic pumps (including a multiple pump unit), syringe pumps, electrically and pneumatically actuated valves, and an assortment of spectrophotometric and electrochemical detectors. Details of the interface circuitry are given where appropriate. To demonstrate the utility of the system, an automatically generated response surface is presented for the flow injection determination of iron(II) by its reaction with 1,10-phenanthroline.*

Continuous flow methods of analysis, such as flow injection analysis (FIA) [1,2] and air-segmented continuous flow analysis (ASCFA) [3], have become well-established analytical tools. These methods are characterized by their versatility, high throughput, precision and simplicity, which make them ideal for routine analyses in many fields. One difficulty with continuous flow methods is that performance factors such as sensitivity and selectivity are difficult to predict because of the complex interaction of flow dynamics and reaction kinetics. For this reason, empirical optimization methods such as simplex optimization are often used in the course of method development [4]. Such development procedures typically require the manual preparation and mixing of solutions. However, Betteridge *et al.* have shown that use of a computer-controlled continuous flow system can greatly reduce the time and effort required to optimize methods [5]. By placing the computer in control of hardware such as pumps, valves, and detectors, it is possible to automate solution preparation and measurement of the analytical response, and so remove the operator from the optimization loop. This improves the speed and reliability of the system and frees the chemist for more important tasks.

The use of programmable continuous flow instrumentation has other advantages. Chemical response surfaces can be mapped as a function of several variables with minimal operator intervention [6]. This is important, as such studies normally involve complete factorial designs with a large number of experiments. With an automated system it is also possible to carry out complex FIA experiments with greater ease. These include experiments utilizing concentration gradients [7], merging zones [8], and flow reversals [9], where reproducible timing is critical.

† Present address: Department of Chemistry, Dalhousie University, Halifax, Nova Scotia, Canada B3H 4J3.

‡ Author to whom correspondence should be addressed.

*Table 1. Elements of the automated continuous flow system.*

| *Category* | *Description* |
|---|---|
| Pumps | (a) Multiple peristaltic pump unit (5 independent pump heads, stepper motor driven) |
| | (b) Stand-alone peristaltic pumps (2) |
| | (c) Syringe pumps (2) |
| Valves | (a) Pneumatically actuated six port valves (4) |
| | (b) Electrically actuated six port valves (3) |
| Detectors | (a) Simple LED-based photodetectors |
| | (b) Dual beam filter photometer |
| | (c) Diode array spectrophotometer |
| | (d) pH electrodes |

Since its initial development, the automated continuous flow methods development system of Betteridge *et al.* [5] has undergone many changes. The availability of this programmable hardware made possible more and more sophisticated experiments, which in turn demanded greater computing capabilities. For this reason, the Commodore PET computer which was originally used to control the system has been replaced with an IBM PC-AT compatible computer. This has necessitated some changes in hardware and software. In addition, there was a need for greater versatility in the hardware available for the wide range of experiments which could be carried out. To meet this need, the system has been expanded and is now capable of controlling additional continuous flow components.

This paper describes in detail the controlling hardware of the current flow injection development and optimization (FIDO) system. This description includes elements of the original system of Betteridge *et al.* [5], not previously described in detail, as well as components of the modified system.

### System components

Table 1 lists the continuous flow system components which are part of the current FIDO system. The software is capable of controlling and/or monitoring three types of

0142–0453/89 $3.00 © 1989 Taylor & Francis Ltd.

pumps, two types of valves, and four types of detectors. Specific descriptions of the major system components follow.

*Computer*

The heart of the FIDO system is the IBM PC-AT compatible computer (Nora Systems, Vancouver, BC, Canada) which controls all of the continuous flow system components. This is done through the use of three interface boards: (1) an IBM data acquisition and control adapter (DACA) board (Mendelson Electronics, Dayton, OH, USA); (2) an HPIB (IEEE-488) interface card (Hewlett-Packard, Palo Alto, CA, USA); and (3) two serial ports. The FIDO software, which facilitates calibration, configuration specification, control, optimization and response surface mapping, is written in Microsoft Quick BASIC version 4.0 and currently consists of about 200 Kbytes of source code, excluding external libraries.

*Pumps*

Most applications will not require all of the pumps which can be controlled by the FIDO system, but the variety of available units makes it possible to select a configuration that is appropriate for a given application. The multiple peristaltic pump unit is a modification of that constructed by Betteridge *et al.* [5]. The unit consists of five independent pump heads (Ismatec, Zurich, Switzerland, type mini-micro 2/6) housed in a 19 inch rack and driven by stepper motors (Impex type). Driver boards (McLennan, Camberley, Surrey, UK, type EM127) provide the necessary phasing and current forcing circuitry to drive the stepper motors. The speed of these pumps can be varied from 0 to 76 revolutions per minute, with a resolution of 1 part in 255. One of these pumps is reversible.

Because the speed resolution of the multiple pump unit is not adequate for some applications, the system is also capable of controlling two higher precision peristaltic pumps (models C4V and C6V, Alitea Inc., Medina, WA, USA) which have a resolution of 1 part in 4095. The range over which this resolution is obtained can be adjusted on the front panel of the pump, and the rotation of the pump heads at low speeds is much smoother than for the multiple pump unit. The direction of these pumps can also be controlled by the computer.

Finally, for applications where an accurate knowledge of the volumetric flow rate setting is required, or pump pulsations must be minimized, two syringe pumps may be used. These are dual syringe units (model 22 infusion pump, Harvard Apparatus, South Natick, MA, USA) which are capable of reversible operation.

*Valves*

Both electrically and pneumatically actuated valves can be accommodated by the modified FIDO system. The original system used only a single motor driven valve. While electrically actuated motor driven valves have the advantage of requiring no compressed gas supply, they generally respond more slowly than those with pneumatic actuation. The current system permits more than one valve of either type to be used. Three pneumatically actuated six-port valves (type 50 valves with model 5701 actuators and model 7163 solenoid valves, Rheodyne Inc., Cotati, CA, USA) are mounted in a single valve controller unit, and an additional single valve unit is run by a separate controller. The electrically actuated six-port valves (model 86916 with drivers, Hamilton Valve Co., Reno, NV, USA) are mounted individually on their controllers.

*Detectors*

Three different spectrophotometric detectors are available to meet the varying demands of continuous flow experiments. The first is based on a previous design by Sly *et al.* [10] and was used in the original system [5]. The design incorporates an LED source and phototransistor detector mounted transversely on transparent tubing. This design is simple, inexpensive, and quite effective for many applications, but the short pathlength, broad source spectrum, and limited wavelength options restrict its capabilities. An alternative is a detector based on the design of Patton *et al.* [11]. This detector has a quartz halogen source with interference filters and an axial geometry, ensuring a greater range of applications. Finally, a diode array spectrophotometer (model 8452A, Hewlett-Packard, Palo Alto, CA, USA) can be used for maximum flexibility. Although this instrument is generally too expensive for routine applications, it is ideal for development work and for specialized experiments.

The FIDO system can also accommodate several electrochemical detectors. For pH measurements two 50 μl flow-through combination electrodes (model GK743500B, Radiometer America, Westlake, OH, USA) are currently available for use. An amplified and compensated voltage output can be provided by a standard pH meter (model 119, Fisher Scientific, Vancouver, BC, USA), or a dual electrode amplifier constructed as part of the FIDO system may be used. The dual electrode amplifier will also accommodate ion selective electrodes.

### Interface circuitry

This section describes details of the interface circuitry used to control the various elements of the FIDO system.

*System bus*

The original FIDO system was interfaced to a Commodore Pet through ports located within the ROM expansion area of the microcomputer's memory. When the system was modified for control by an IBM PC-AT compatible, it was clear that some aspects of the original interface would have to be changed. In making these changes, several goals were established: (1) to maintain, as much as possible, compatibility with existing system components; (2) to develop an improved interface which could be easily adapted to future changes in computer hardware; (3) to remove, as much as possible, dependence on external interface elements and instead rely on more standardized interface boards; and (4) to allow for easy future expansion.

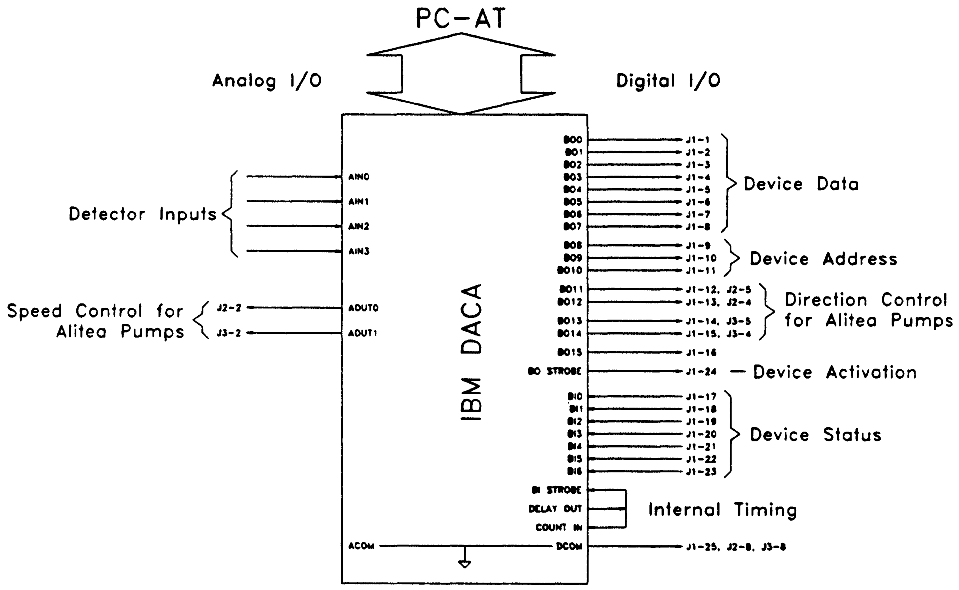

*Figure 1. Configuration of FIDO system interface bus.*

To meet the last three of these goals, it was decided to employ a fairly standard interface board. The IBM data acquisition and control adapter was selected with 16 digital input and output lines, a four channel, 12-bit A/D converter, and two 12-bit D/A converters. There are other boards, or combinations of boards available that also meet or exceed these requirements. The two additional boards incorporated into the FIDO system were readily available IEEE-488 and RS-232 interfaces. These were necessary to communicate with the diode array spectrophotometer and syringe pumps, respectively, and may be useful in the future for communications with devices such as balances and robotic facilities.

Certain components of the automated continuous flow system, such as the multiple peristaltic pump unit and the valves, require interfaces external to the computer. The use of a centralized interface unit not only increases the complexity of the system, but also makes it difficult to establish automated continuous flow systems on other computers without entirely duplicating the interface. To avoid this problem, our aim has been either to eliminate external interfaces or make them self-contained in the device. This makes for much better adaptability.

For those devices which do require external interfaces, a simple bus protocol was established that allows for system expansion. The configuration of the IBM DACA used for this system is shown in figure 1. The first eight digital output lines (BO0 to BO7) are used to provide data to the external interface which is addressed with the next three lines (BO8 to BO10). The output datum is received by the device when the address is valid and the BO Strobe handshake line goes high. This allows up to eight external independently addressable interfaces to be used. Digital outputs BO11 to BO14 are used to provide direction control for the Alitea pumps. This control could have been accomplished using an external interface, but this way the pumps can be controlled directly and implementation on other computers is not hindered. The remaining digital output line, BO15, is currently unused.

Digital inputs BI0 to BI6 are used to return status from external interfaces. To facilitate future expansion, external interfaces use tristate outputs which become active only when the device is addressed. This allows multiple devices to use the same status lines. Digital inputs BI7 to BI15 are currently unused. The output of counter 1 (Delay Out) of the 8253 counter/timer on the DACA board is connected to the BI Strobe input. The output of this counter is also connected to the input of counter 2 (Count In). This configuration provides convenient timing for data acquisition and other applications.

The four analog-to-digital converter (ADC) inputs can be connected directly to detector outputs in most cases. Differential inputs are used and the voltage range for 12-bit conversion is $-5V$ to $+5V$. Programmable gain is not available with the DACA board, but other data acquisition boards provide this feature as well as a great number of inputs if these features are important. The two digital-to-analog converter (DAC) outputs are used to control the speed of Alitea pumps and have a 12-bit resolution in the range 0 to 10V.

In addition to the standard BNC connectors used for analog inputs, there are three connectors from the DACA board. Connector J1 is a 25-pin D-connector which goes to external interfaces. Connectors J2 and J3 are 15-pin D-connectors which provide control signals for the two Alitea pumps.

### *Pumps*

#### *Multiple peristaltic pump unit*

In the original paper of Betteridge *et al.* [5], only a brief description of this unit was given. In this section, details of the interface are provided. A circuit diagram of the interface is given in figure 2. Timing circuitry is shown for only one of the pump units (five pumps are available).

The pump interface is assigned two addresses on the system bus, one for pump selection and one for speed

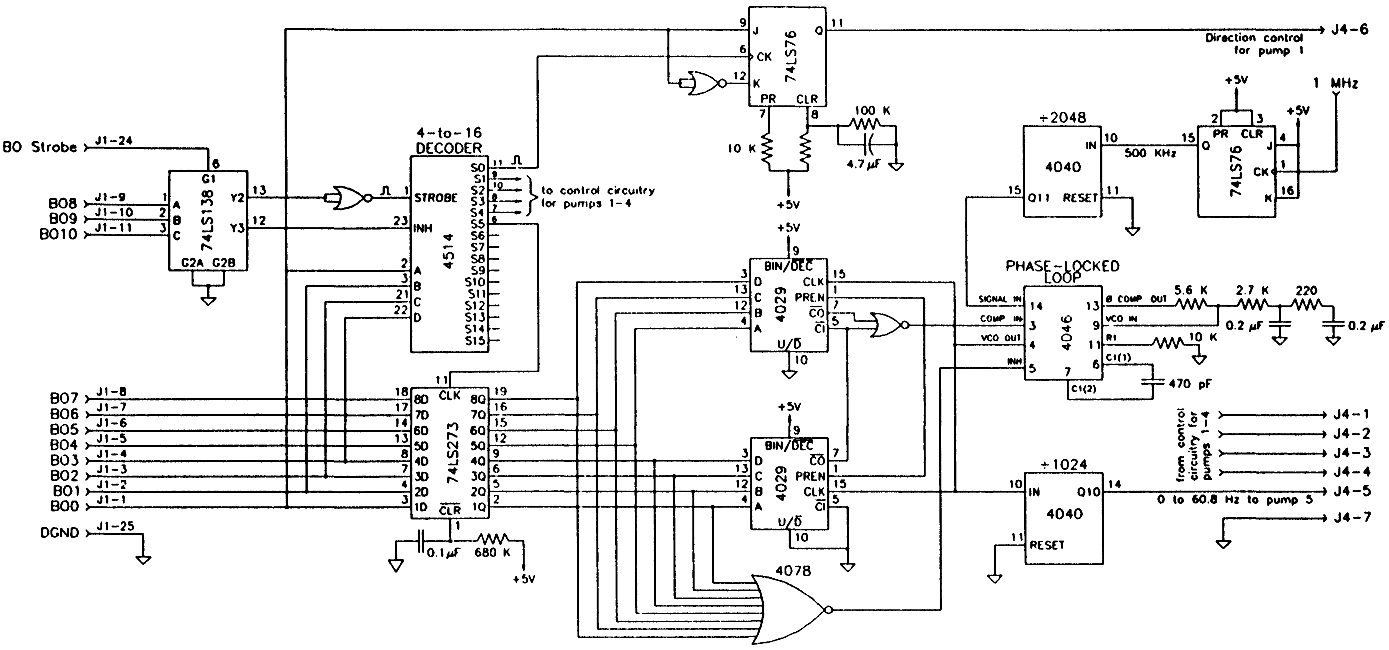

*Figure 2. Circuit diagram for multiple peristaltic pump unit interface. Timing circuitry is only shown for pump five.*

selection. The sequence for programming the pump speed is as follows: first a pump is chosen by selecting device address two on the address bus (BO8 to BO10), putting the pump number (1–5) on the data bus (BO0 to BO7), and toggling the BO Strobe line. This latches the pump number into the 4-to-16 decoder (4514B). The output of the decoder remains low, however, since the INH input is still high. The next step is to select device address three on the address bus, place the pump speed (0–255) on the data bus, and once again toggle BO Strobe. This causes the INH line to the 4-to-16 decoder to go momentarily low, toggling the appropriate output (S1 to S5) and locking the pump speed into the corresponding 8-bit latch (74LS273). The outputs from this latch are the preset inputs to two cascaded 4-bit binary counters (4029B), which act as a variable frequency divider. The input to the frequency divider is provided by the voltage controlled oscillator output (VCO) of a phase-locked loop (4046B), whose input is a reference signal of 244 Hz. This arrangement has the effect of multiplying the input frequency by the divider setting to obtain the output frequency (VCO). Additional logic disables VCO when a speed of zero is specified. Thus, the frequency at VCO will vary from 0 Hz to (255 × 244) Hz in uniform increments. This output is further divided by a factor of 1024 by a binary counter (4040B) before being sent to the stepper motor control circuitry. The control signal varies from 0 to 61 Hz and each pulse corresponds to a 7·5° rotation of the motor. The actual stepper motor drive circuitry is commercially available, so details will not be given here.

The timing circuitry shown in figure 2 for pump 5 is identical to that employed for the other four pumps. Only one decoding circuit is needed. Direction control for pump 1 is available by selecting pump 0 in the pump addressing cycle. In the speed selection cycle, BO0 provides the pump direction input (1 = forward, 0 = reverse). The direction control is latched into a JK flip-flop (74LS76) and sent directly to the stepper motor control circuitry. In the original design, this direction signal controlled all of the pumps, but the design has been modified so that pumps 2 to 5 are unidirectional. This modification provided greater versatility. In principle, it is possible to control the direction of all of the pumps independently, but this would have required modification of the original design to provide extra latches and additional lines on connector J4.

The additional outputs (S6 to S15) of the 4-to-16 decoder are currently unused, but allow for future expansion. They may be used to trigger external devices (e.g. valves) or to accommodate an additional multiple pump unit. In its current state the multiple peristaltic pump unit has performed without problems for many hours and is the workhorse of the FIDO system.

### *Alitea peristaltic pumps*

Control of the Alitea peristalic pumps is facilitated through two external digital connections for direction control, and an additional contact for a speed control voltage of 0 to 10 V. This voltage is provided by the output of a 12-bit DAC, and provides a speed resolution of 1 part in 4095. The maximum speed for this voltage range is set on the front panel of the pump. Two DACs are currently available with the IBM DACA board, allowing control of two pumps. This number can easily be increased by adding additional DACs.

### *Syringe pumps*

The Harvard Apparatus syringe pumps are controlled through an RS-232 communications interface. A simple command protocol is used to control flow rate and pump direction, and obtain status information. The PC-AT compatible computer currently used allows up to four

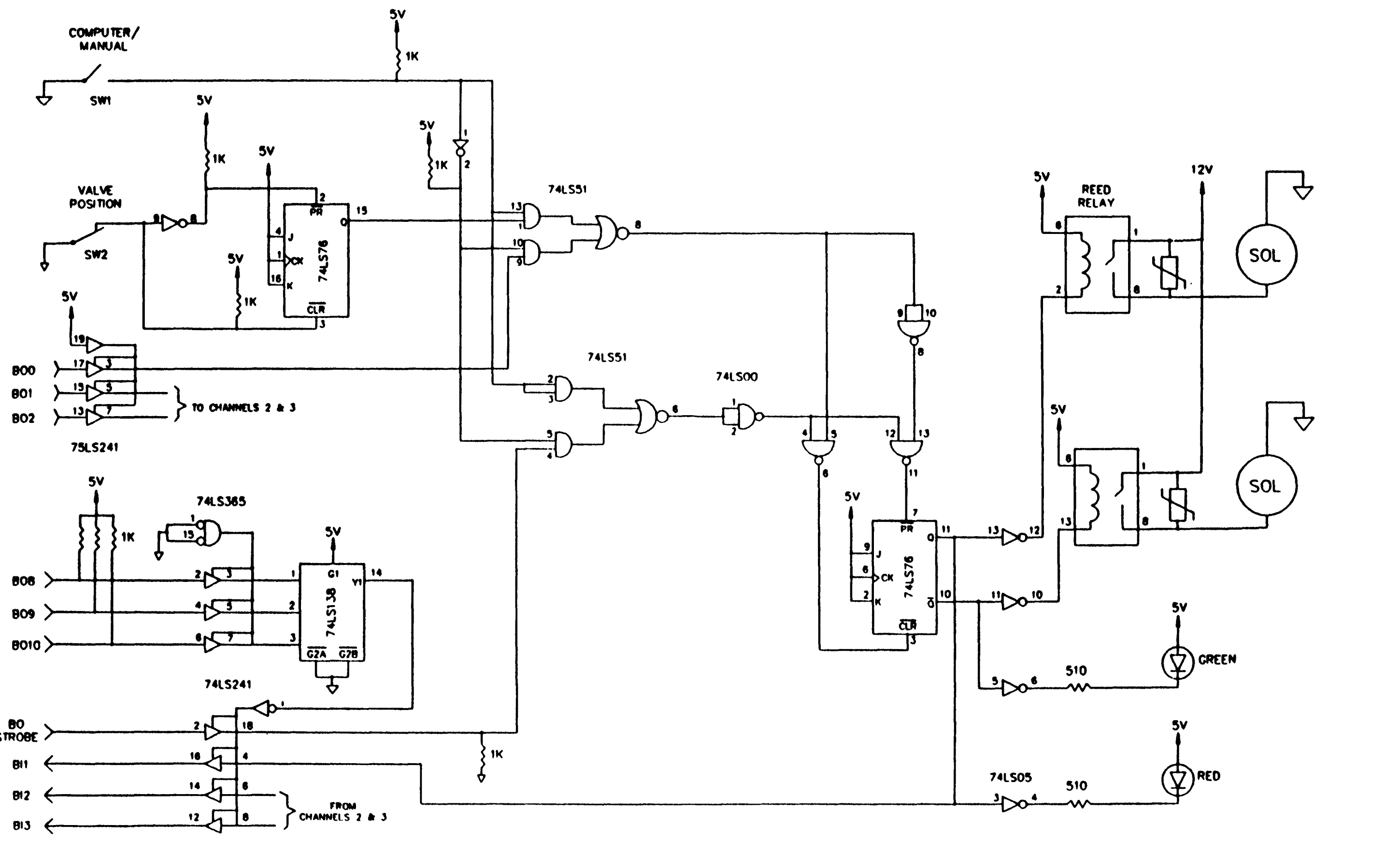

*Figure 3. Circuit diagram for one channel of the three-channel pneumatic valve controller.*

serial ports to be employed for the control of four syringe pumps. Further expansion is possible with commercially available boards or external multiplexers.

### *Valves*

#### *Pneumatically actuated valves*

The current FIDO system is capable of controlling four pneumatically actuated six-port valves. Three of these valves are mounted in the same controller unit to accommodate special FIA experiments, such as those involving merging zones, which sometimes require valves to be in close proximity. The other is mounted independently with its own controller. The design for both controllers is essentially the same. The circuit diagram for the three-channel valve controller, showing decoding and control circuitry for valve 1, is shown in figure 3.

The valve actuators used for this system require positive pressure actuation in both directions, so two solenoid valves (which should always be in opposite states) are needed. These require a current of 500 mA each and are driven by reed relays (PRMA1A05). A metal oxide varistor (18V DC) is used on the output of each relay to suppress transients on the power supply. The relays are driven by complementary outputs of a JK flip-flop through open-collector inverters.

The controller allows both manual and computer control on each channel. In manual mode, switch 1 is open and inputs are provided directly by switch 2. The JK flip-flop (74LS76) at the switch 2 input acts as a debouncing circuit. All succeeding gates are enabled and the JK flip-flop controlling the solenoid valves is either preset or cleared according to the input at switch 2.

In computer control mode, switch 1 is closed, and switch 2 is disabled by the upper AND-OR-INVERT (AOI) gate (74LS51). The controller now responds to the state of BO0, but only if the device is addressed and BO Strobe is high. Address decoding is performed by the 74LS138 and the device address is currently set to 1, although this is easily changed. The decoder output controls a tri-state buffer (74LS241) to permit BO Strobe to reach the controller circuitry, and allow the valve position status lines to appear on the bus. When the device is addressed and BO Strobe is high, BO0 provides the input to the controlling flip-flop. This input is latched when BO Strobe goes low. To ensure correct operation when the computer is disconnected, a pull-down resistor is connected to the BO Strobe input. The status lines (BI1 to BI3) are valid whenever the device is addressed and are in the high-impedance state otherwise so these lines may be used by other devices. Channels 2 and 3 of the valve controller are driven by BO1 and BO2. Identical controlling circuitry is needed, but additional address decoding is unnecessary.

For the single channel valve controller, some modifications were made to reduce the component count. These included: (1) replacement of the 74LS138 decoder with discrete logic; (2) elimination of the 74LS365 line driver;

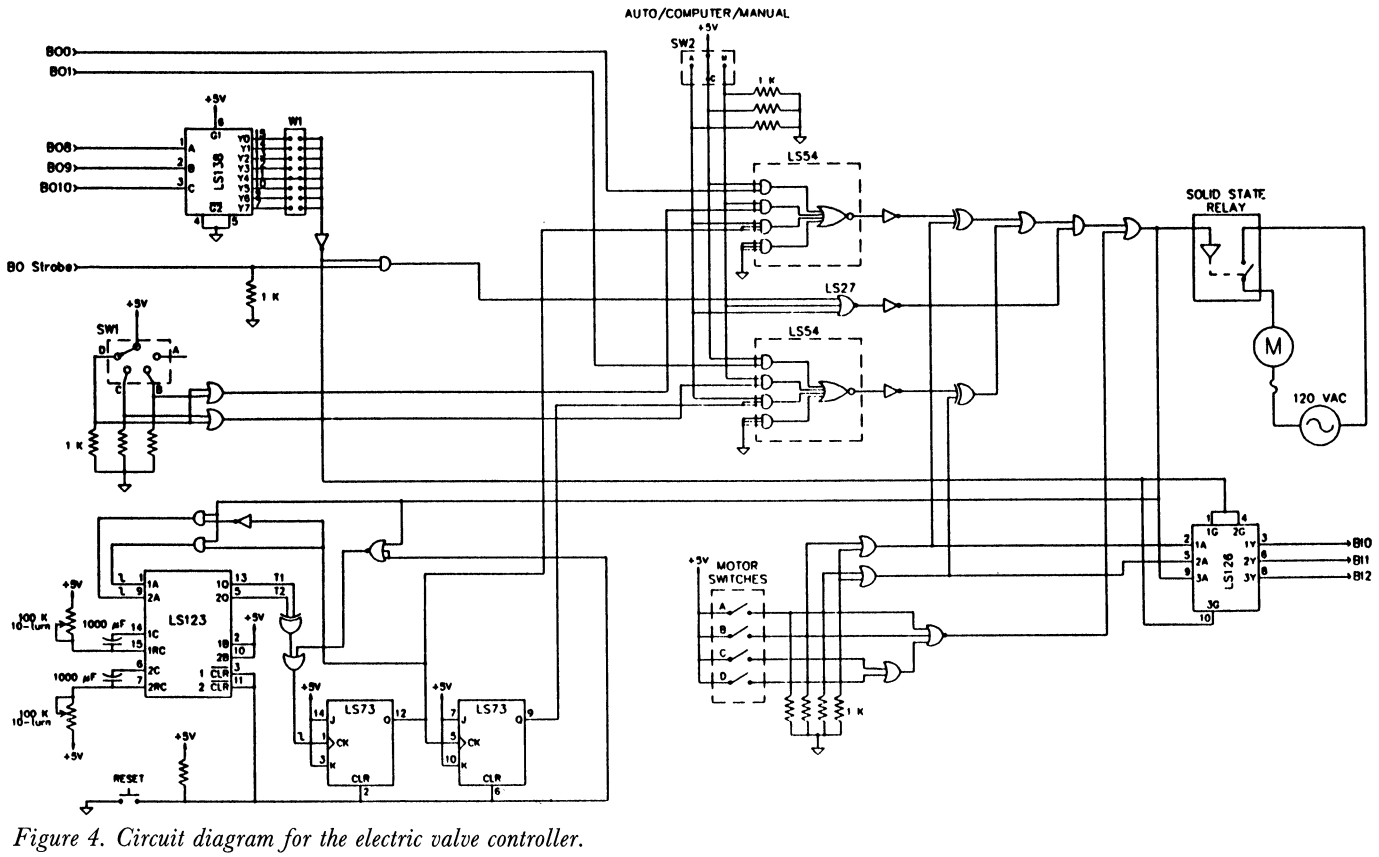

*Figure 4. Circuit diagram for the electric valve controller.*

(3) replacement of the 74LS241 line driver with the smaller 74LS125; and (4) use of a double pole relay rather than two single pole relays. In addition, the logic was powered by a 9V battery and 5V regulator to provide better isolation from the solenoid power supply. This single channel controller could be implemented with only six chips, including the relay.

Both of the valve controller units have proved to be rugged and effective. Earlier designs attempted only temporary solenoid actuation, as this is really all that is required. This increased the chip count and was more prone to problems from noise in the system.

*Electrically actuated valves*

The electrically actuated six-port valves are driven by unidirectional motors with microswitches to indicate when each of the four positions (90° increments) is reached. A circuit diagram for the valve controller is given in figure 4. The same controller could also be used for a four-port selector valve.

The position to which the valve is to be driven is specified by a binary value between 0 and 3. This is provided on the left-hand side of the circuit diagram and comes from one of three sources: a manual switch, a circuit for automatic timing, or the computer. The source of the control is specified by switch 2, which is used to enable appropriate inputs of the two AOI gates (74LS54) used.

In manual mode, the position of the valve is specified by switch 1. The setting of this four-position switch is decoded to binary with two OR gates (74LS32). These two outputs pass through the two AOI gates to two XOR gates (74LS86), which compare the requested position with the binary value of the current position decoded from the four microswitches on the motor. The XOR outputs will activate the motor through a solid state relay if the two positions are not equal. Additional logic between the XOR outputs and the relay input ensures that: (1) the valve position does change when switching from manual to computer mode, and (2) the motor does not stop while the valve is between positions.

In automatic timing mode, the two binary outputs which specify the motor position are provided by timing circuitry in the lower left-hand corner of the circuit diagram. The dual JK flip-flop (74LS73) acts as a two-bit binary counter and provides these binary outputs. Asymmetric timing is provided by the dual monostable multivibrator (74LS123). The two timing periods are adjustable from potentiometers on the front panel and are alternately triggered when the motor stops moving. A reset switch is also provided to initialize the valve sequence. Additional logic on the reset circuitry ensures that the monostable will continue to receive trigger pulses even if the reset switch is depressed for an extended period. The binary outputs provided by the automated timing circuit are processed in the same manner as for the manual circuitry.

For computer control, the two binary outputs which specify the valve position are provided on the system data bus. In this mode, a two-input AND gate (74LS00) and a three-input NOR gate (74LS27) prevent the motor from

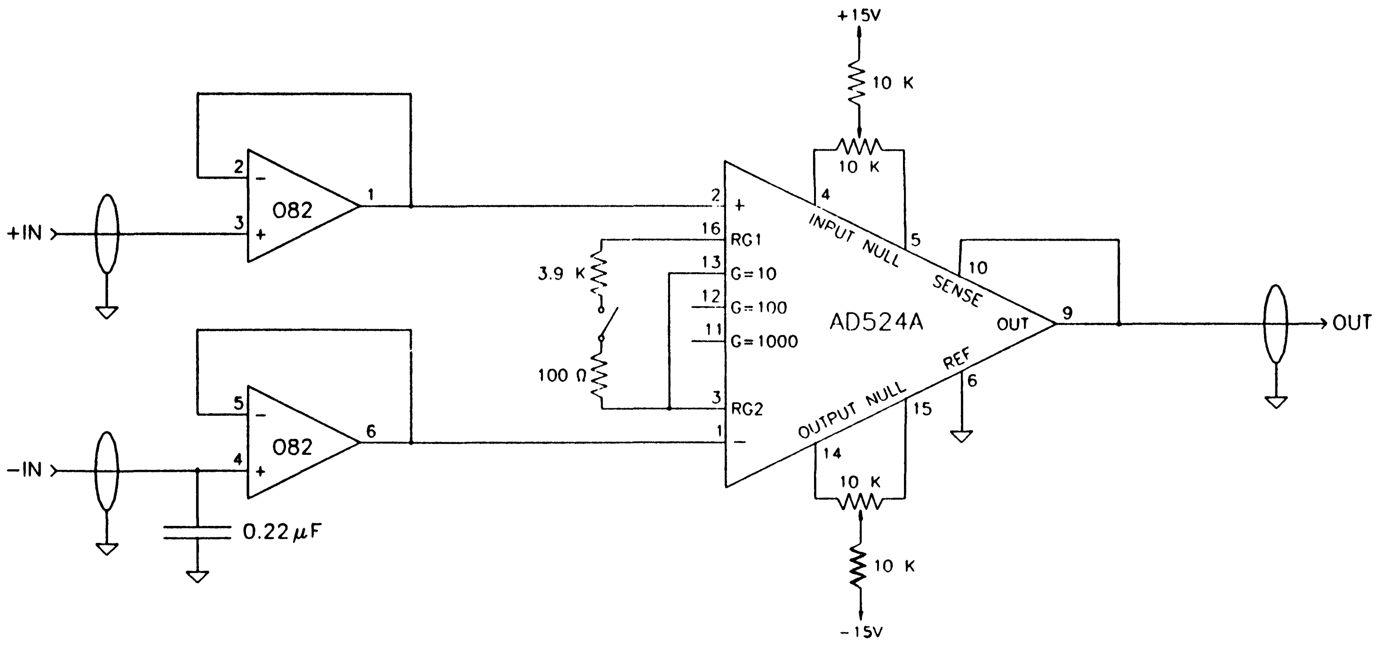

*Figure 5. Differential electrode amplifier circuit.*

moving until the controller is enabled, i.e. until the device address appears on the address lines and BO Strobe goes high. The controller must remain enabled until the motor reaches the desired position. A 3-to-8 decoder (74LS138) is used to decode the jumper-selectable address. A tristate buffer (74LS126) returns the controller status on the system bus whenever the address is selected. Two status bits indicate the motor (valve) position; a third is set if the motor is currently activated.

*Detectors*

*Spectrophotometric detectors*

The two simpler type of photometric detectors which are part of the current FIDO system were constructed in our laboratory. The designs for these have been reported elsewhere [10,11] and will not be repeated here. In each case, the output of the detector is connected directly to one of the ADC inputs of the IBM DACA board. The system can facilitate multiple detectors. Control and monitoring of the diode array spectrophotometer is possible through an IEEE-488 interface card. IEEE-488 drivers provided by Hewlett-Packard permit direct communication with the instrument from the FIDO software.

*Electrochemical detectors*

Capacity for pH and ion selective electrode detection on the FIDO system is provided in two ways. First, a separate pH/mV meter with amplified outputs may be employed. The Fisher meter currently used has this capability, with an amplification of 0·1 V per pH unit. This may be connected directly to an ADC input of the IBM DACA board.

A second means of amplifying the signal from the detector electrodes is to use the electrode amplifiers which were constructed as part of the original FIDO system. These differential amplifiers were intended as a low cost alternative for monitoring multiple detectors. Their design is relatively simple and shown in figure 5. Differential inputs are provided for detector and reference electrodes, and are buffered with high impedance voltage followers (O82). Often, as with combination electrodes, a reference input is not needed and can be connected to common. A capacitor is used on the reference channel input to reduce noise, but is not included at the signal channel where it would increase response time. The buffered differential inputs are fed into an instrumentation amplifier (AD524A). The gain of the amplifier is switch selectable between 10 and 20. The amplifier output is connected to the one of the ADC inputs of the IBM DACA board. Slope and offset compensation for the electrode are not available, but these are not essential and can be performed in software if needed. Currently, there are two electrode amplifiers in the FIDO system, but this number can be easily expanded as the need arises.

### Applications

The system described above has been employed in a wide variety of chemical studies for the purposes of automated optimization and response surface modelling. As an example of a typical case, the flow injection determination of iron(II) by its well known reaction with 1,10-phenanthroline [12] was studied. The manifold used for this study is shown in figure 6. A $10^{-2}$ M solution of 1,10-phenanthroline monohydrate was employed as the reagent. The flow rate of this was varied between 0·1 and 1·0 ml/min, with a distilled water diluent maintained at a flow sufficient to keep the total flow rate of the reagent steam at 1 ml/min. A $10^{-2}$ M solution of hydroxylamine hydrochloride at a fixed flow rate of 0·5 ml/min was employed as a reducing agent for any iron(III) which might be present. This stream was merged with an

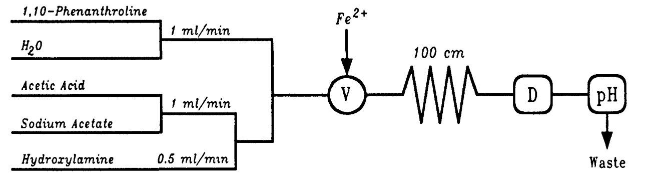

*Figure 6. Manifold employed for the iron(II)/phenanthroline study.*

acetate buffer stream made up of 0·2 M acetic acid and 0·2 M sodium acetate. Although relative flow rates of the buffer components could vary, the total buffer flow rate was maintained at 1 ml/min. The sample was a $10^{-4}$ M solution of iron(II) ammonium sulphate prepared in dilute sulphuric acid (2·5 ml/l). After injection and reaction of the sample, absorbance of the red complex formed was measured at 508 nm. Carrier pH was also monitored with a flow-through combination electrode located downstream from the cell.

To construct a chemical response surface, the flow rates of the phenanthroline and sodium acetate were automatically and systematically varied by the computer, with appropriate adjustment of distilled water and acetic acid diluents. The response, in the form of the peak absorbance for the injected sample, was measured in triplicate by the computer under each set of conditions. In this way, the effect of phenanthroline concentration and pH were examined at a fixed carrier flow rate. A total of 300 experiments were performed over a period of several hours. These required no operator intervention except for filling of solution bottles. The surface showing the effects of carrier pH and phenanthroline concentration in the carrier is shown in figure 7. The surface was generated from the data with the 3-D plotting package SURFER (Golden Software, Golden, CO, USA). It is clear that such surfaces contain a wealth of information for the analytical chemist and the value of an automated apparatus for generating such information will undoubtedly increase with the complexity of the system and the number of variables being considered.

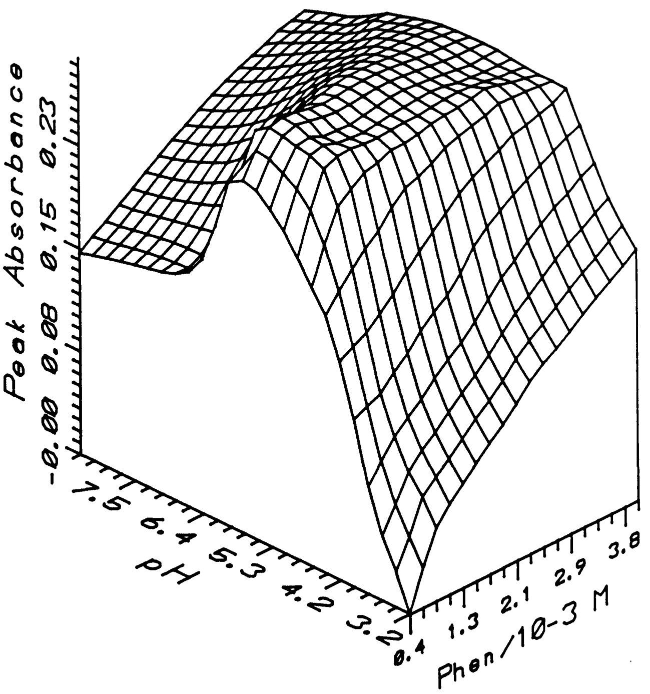

*Figure 7. Response surface showing the effects of carrier pH and 1,10-phenanthroline concentration on the determination of iron(II) by FIA. The response is the peak absorbance for the injected sample.*

### Acknowledgements

The authors gratefully acknowledge the assistance of Joe Sallos and Russ Hamilton of the UBC chemistry electronics shop. This work was made possible through NSERC Grant Nos. 5-80246 and 5-80085, and through an NSERC Postdoctoral Fellowship (PDW). Support was also provided through a UBC University Graduate Fellowship (PMS).

### References

1. Ruzicka, J. and Hansen, E. H., *Flow Injection Analysis,* 2nd edn (Wiley, New York, 1988).
2. Betteridge, D., *Analytical Chemistry,* **50** (1978), 832A.
3. Furman, W. B., *Continuous Flow Analysis* (Marcel-Dekker, New York, 1976).
4. Betteridge, D., Sly, T. J., Wade, A. P. and Tillman, J. E. W., *Analytical Chemistry,* **55** (1983), 1292.
5. Betteridge, D., Sly, T. J., Wade, A. P., and Porter, D. G., *Analytical Chemistry,* **58** (1986), 2258.
6. Shiundu, P. M., Wentzell, P. D. and Wade, A. P., *Talanta* (in press).
7. Horner, J. A., Wade, A. P. and Blades, M. W., *Journal of Analytical Atomic Spectrometry,* **3** (1988), 809.
8. Bergamin, H., Zagatto, E. A. G., Krug, F. J. and Reis, B. F., *Analytica Chimica Acta,* **101** (1978), 71.
9. Betteridge, D., Oates, P. B. and Wade, A. P., *Analytical Chemistry,* **59** (1987), 1236.
10. Sly, T. J., Betteridge, D., Wibberly, D. and Porter, D. G., *Journal of Automatic Chemistry,* **4** (1982), 186.
11. Patton, C. J. and Crouch, S. R., *Analytica Chimica Acta,* **179** (1986), 189.
12. Day, R. A., Jr. and Underwood, A. L., *Quantitative Analysis,* 5th edn (Prentice-Hall, Englewood Cliffs, NJ, 1986).

